# Incorporation size of lymph node metastasis focus and pre-ablation stimulated Tg could more effectively predict clinical outcomes in differentiated thyroid cancer patients without distant metastases

**DOI:** 10.3389/fendo.2023.1094339

**Published:** 2023-03-21

**Authors:** Jiahao Xie, Pan Chen, Jing Wang, Xiaoqin Luo, Jiaxin Luo, Xiaoli Xiong, Chunyan Li, Liqin Pan, Juqing Wu, Huijuan Feng, Wei Ouyang

**Affiliations:** Department of Nuclear Medicine, The Zhujiang Hospital, Southern Medical University, Guangzhou, Guangdong, China

**Keywords:** differentiated thyroid cancer, lymph node metastasis, pre-ablation stimulated Tg, 131I therapy, prognosis

## Abstract

**Background:**

The size of lymph node metastasis (LNM) and pre-ablation stimulated Tg (ps-Tg) were key predictors of clinical prognosis in differentiated thyroid cancer (DTC) patients, however, very few studies combine the above two as predictors of clinical prognosis of DTC patients.

**Methods:**

Persistent/recurrent disease and clinicopathologic factors were analyzed in 543 DTC patients without distant metastases who underwent LN dissection, near-total/total thyroidectomy, and radioiodine ablation.

**Results:**

In the multivariate analysis, size of LNM, ps-Tg, and the activity of ^131^I significantly correlated with long-term remission. The optimal cutoff size of LNM 0.4 cm-1.4 cm (intermediate-risk patients) and >1.4cm (high-risk patients) increased the recurrence risk (hazard ratio [95% CI], 4.674 [2.881-7.583] and 13.653 [8.135–22.913], respectively). Integration of ps-Tg into the reclassification risk stratification showed that ps-Tg ≤ 10.1 ng/mL was relevant to a greatly heightened possibility of long-term remission (92.2%–95.4% in low-risk patients, 67.3%–87.0% in intermediate-risk patients, and 32.3%–57.7% in high-risk patients).

**Conclusion:**

The cutoff of 0.4 cm and 1.4 cm for a definition of size of LNM in DTC patients without distant metastases can reclassify risk assessment, and incorporating ps-Tg could more effectively predict clinical outcomes and modify the postoperative management plan.

## Introduction

In recent decades, differentiated thyroid cancer (DTC), specially papillary thyroid cancer (PTC), is continually metastasizes to the cervical lymph nodes(LN), the occurrence of lymph node metastases (LNM) in PTC has been reported to range from 31.5% to 50.0% (1–4). Therefore, further attention has been paid to accurate evaluation of the risk stratification in patients with DTC. Recently, the 2015 American Thyroid Association (ATA) Initial Risk Stratification System based on LNM has been proposed for assessing the chance of having poor prognosis during follow-up (5, 6). Nevertheless, based on clinical experience, the size of LNM is commonly distributed between 0.2 cm and 3 cm. Therefore, the 2015 risk stratification system may not precisely predict the clinical prognosis.

The size of LNM is defined as a focus full of thyroid cancer metastases in the LNM with the largest dimension. Emerging literature has demonstrated that the size of LNM is an independent risk factor of persistent/recurrent disease in DTC patients. As the size of LNM increases, it is more significantly associated with poor prognosis (7–15). Recent writing has reported that pre-ablation stimulated Tg (ps-Tg) measured under levothyroxine withdrawal just earlier radioiodine (RAI) ablation is an independent risk factor of persistent/recurrent disease, the ps-Tg (≤10.1 μg/L) is greatly connected with long-term remission in DTC patients (13, 16).

The size of LNM and ps-Tg were key predictors of clinical prognosis in DTC patients, however, very few studies combine the above two as predictors of clinical prognosis of DTC patients. This research was undertaken to find out the optimum cutoff size of LNM and to incorporate size of LNM and ps-Tg to accurately assess clinical prognosis in patients with DTC.

## Materials and methods

### Data source

We retrospected the electronic medical records of DTC patients from July 2014 to June 2017 at the Department of Nuclear Medicine at Zhujiang Hospital, Southern Medical University, after getting of approval by the local Institutional Review Board.

### Patients

**Figure 1:**
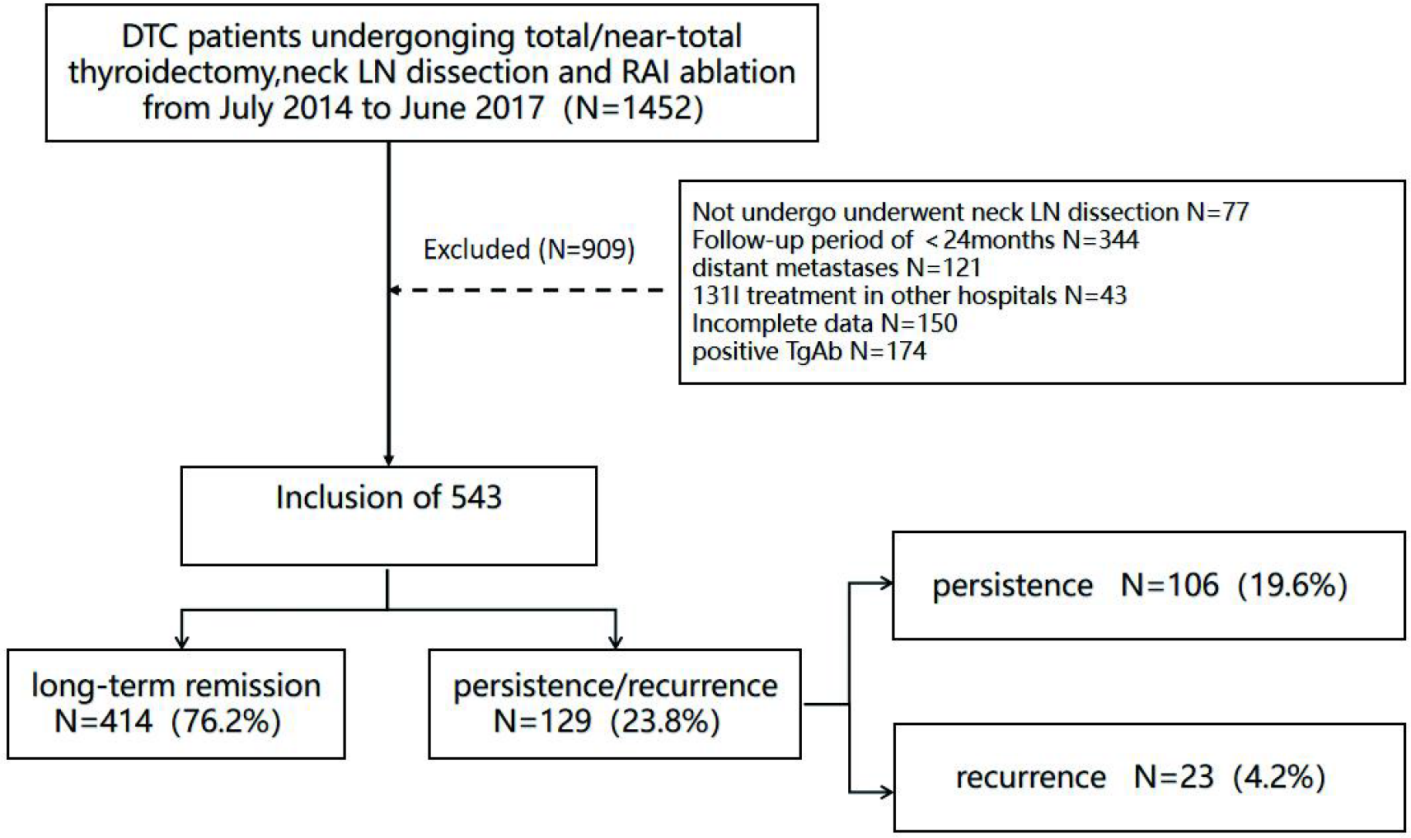
Initial patients’ selection, occurrence of persistence/recurrence during follow-up.

The patients who underwent cervical LN dissection, near-total/total thyroidectomy and then conduct the first RAI ablation in our hospital (17) were ruled out if they meet the following standards (1): patients had distant metastases upon surgery or in initial RAI ablation, (2) patients had missing data or incomplete data on size of LNM, (3) patients had previous RAI ablation in other hospitals, (4) patients had positive thyroglobulin antibody (TgAb) (> 115 IU/mL), (5) patient follow-up time was < 24 months after the first RAI ablation (**Figure 1**).

The data was reviewed including patient demographics, surgery, and pathology reports. The pathologic TNM classification was based on the American Joint Commission on Cancer (AJCC), 8th edition.

### Size of LNM and Tg measurements

The size of LNM is defined as a focus full of thyroid cancer metastases in the LNM with the largest dimension observed in multiple serial sections using microscope. The pathology slides were analyzed and measured the size of metastatic foci in each LN by An experienced pathologist (7).

Roche Cobase 801 electrochemiluminescence instrument was utilized for measurement of Tg (measuring range: 0.04–5000 μg/L). All ps-Tg was measured underlevothyroxine withdrawal just earlier RAI ablation in the same laboratory in our hospital.

### Treatment and follow-up

Before RAI ablation, patients were informed to obey a low-iodine dietary for 3–4 weeks and levothyroxine withdrawal for 3 weeks; this was prolonged for 1 week if TSH levels did not reach 30 μIU/mL. An empiric RAI administered activity was used to decide the activity of 131I, using 1.85–3.70 GBq for thyroid remnant ablation and 3.70–7.40 GBq for postoperative residual cervical LNM.

After the initial RAI ablation, follow up every 3-6 months for the first 2 years. When achieving stable disease, follow up every 6-12 months. Patients were regularly followed-up with *via* measurement of Tg, thyroglobulin antibody (TgAb), and neck ultrasound during thyroid hormone therapy, using the 2009 ATA guidelines (18). Additional imaging methods including 18F-FDG PET/CT, 131I whole-body scans(WBS), chest and mediastinal computed tomography were performed when suppressed Tg (rTG) was >1 μg/L or when suspicious loco-regional/distant metastases were detected.

Incomplete biochemical or structural response within one year of the initial RAI ablation was considered persistence disease. Meanwhile, patients who were earlier considered as having significant effect to therapy was defined as recurrence disease, when they show functional, structural, or biochemical evidence of disease. For the above patients, further treatment plans including repeated surgery, extra RAI therapy, and other therapies were taken by the attending physician.

### Clinical outcomes evaluation and definition

A total of data locked in June 2022 were utilized to determine clinical outcomes. At final follow-up, patients were considered to have long-term remission if they had a rTG < 1 μg/L, no observable TgAb, and no structurally identifiable evidence. Patients were considered as having persistent/recurrent disease if they had a rTG > 1 μg/L, stimulated Tg > 10 μg/L, or any evidence of disease on functional imaging (18F-FDG PET/CT, 131I WBS), structural imaging (ultrasonic, CT, or MRI), or biopsy-proven disease. Patients with persistent/recurrent disease were classed as having either structural evidence of disease on imaging or biochemical evidence of disease (rising TgAb, rTG > 1 μg/L, or stimulated Tg > 10 μg/L without a structural evidence of disease). And patients with structural evidence of disease were fulfilled the undermentioned standards (1): highly suspicious focus on the neck ultrasound (short axis > 5 mm, number, rounded shape, irregular margins, loss of the fatty hilum, enlarged cortex, heterogeneous parenchymal echotexture, hypoechoic parenchymal echogenicity, calcifications, cystic change or necrosis, disorganized peripheral vascularity during follow-up), (2) positive cytology/histology, or (3) findings on 18F-FDG PET/CT, 131I WBS, or other imaging highly suspicious of metastatic evidence (11, 13, 16, 19–21). Disease free survival (DFS) was defined as the period of time among the discovery of persistent/recurrent disease and first operation.

### Statistical analysis

Statistical analysis was performed using SPSS software (version 26.0). Categorical data were presented as numbers and percentages. Qualitative parameters were analyzed using chi square test or Fisher’s exact test and are expressed as frequencies and percentages. Continuous data were presented as the mean ± SD or the median (range). A P value < 0.05 was considered statistically significant. The X-tile software was used to acquire the optimum cut-off for the size of LNM to predict persistent/recurrent disease (22). Multivariate analysis was done for variables with a P value < 0.05 in the univariate analysis to determine the statistical risk factors associated with poor prognosis. Curves of DFS were fabricated using the Kaplan-Meier method, and log-rank tests were used to assess the differences in DFS among the risk stratification. Missing data were handled using complete case analysis.

## Results

### Patient characteristics

Of the 1,452 consecutive DTC patients who underwent near-total/total thyroidectomy administered in our department during the study, 77 patients were excluded because they did not undergo neck LN dissection, 344 patients were excluded because they had <24 months of follow up, 121 patients were excluded because of distant metastases upon surgery or initial RAI ablation, and 43 patients were excluded because they had received previous 131I treatment at other hospitals. In addition, 150 patients were excluded due to missing data or incomplete data on size of LNM (missing pathology reports: N = 32, incomplete data on size of LNM: N = 118), and 174 patients were ruled out due to the positive TgAb (>115 IU/mL). Finally, 543 patients with DTC were involved in the final cohort (**Figure 1**).

The median of the primary tumor size was 1.5 cm (range: 0–9.0 cm). Patients with positive LN was 494 (91.0%), with a median number of positive LNs of 5 (range: 0–79). The median size of LNM was 0.4 cm (range: 0–5.0 cm). The median 131I-administered activity was 5.55 GBq (range: 1.85–22.94 GBq). The number of patients with multiple RAI ablation was 101 (18.6%) (**Table 1**).

**Table 1:** Characteristics of the study patients (n = 543).

Characteristics	Value
No. of patients	543
Sex Male Female	191(35.2%) 352(64.8%)
Age at diagnosis (y), median (range) <55 ≥55	39(6-76) 481(88.6%) 62(11.4%)
Extent of lymph node dissection Central lymph node dissection Lateral lymph node dissection Central and lateral lymph node dissection	247(45.5%) 19(3.5%) 277(51.0%)
Histology Classic PTC FV-PTC FTC Aggressive histology	519(95.6%) 9(1.7%) 5(0.9%) 10(1.8%)
Incomplete tumor resection	8(1.5%)
Gross ETE	87(16.0%)
BMI(kg/m2) <18.5 18.5-24 >24	52(9.8%) 287(52.9%) 190(35.0%)
Primary tumor size (cm), median (range)	1.5cm(0–9cm)
Bilateral tumor	177(32.6%)
Multifocal tumor	253(46.6%)
Capsular invasion	325(59.9%)
Invasion of loco-regional tissues or structures	133(24.5%)
Vascular invasion	64(11.8%)
Invade nerves	41(7.6%)
Ta T0 T1 T2 T3 T4 Tx	3(0.6%) 329(62.3%) 93(17.6%) 67(12.7%) 34(6.4%) 2(0.4%)
Na N0 N1a N1b	48(8.8%) 252(46.4%) 243(44.8%)
Initial risk stratification Low risk Intermediate risk High risk	64(11.8%) 405(74.6%) 74(13.6%)
BRAFV600E mutation of primary tumor	366(80.8%)
Number of invaded lymph nodes, median (range) >5 ≤5	5(0-79) 220(40.1%) 323(58.9%)
Size of the largest metastatic LN(cm),median (range)	0.4cm(0–5cm)
Extranodal extension of LN	199(46.1%)
Multiple RAI ablation	101(18.6%)
Cumulative 131I-administered activities(GBq), median (range) <7.40 ≥7.40	5.55(1.85-22.94) 440(81.0%) 103(19.0%)
Ps-Tg (ng/mL), median (range) <10 ≥10	4.47(0.04-1511.20) 360(66.3%) 183(33.7%)
TSH(μIU/mL), median (range) <30 ≥30	63.19(5.26-115.92) 22(4.1%) 521(95.9%)
TGAb (kU/L), median (range)	14.31(10.00-114.60)
Follow-up information Median follow-up (m), median (range)	63 (24-122)
Last status No evidence of disease Persistent structural disease Persistent biomedical disease Recurrent structural disease Recurrent biomedical disease	414(76.2%) 68(12.5%) 16(2.9%) 38(7.1%) 7(1.3%)

PTC, papillary thyroid cancer; FTC, follicular thyroid cancer; FV-PTC, follicular variant PTC; ps-Tg, pre-ablation stimulated thyroglobulin; LN, lymph node; BMI, Body Mass Index; ETE, extrathyroidal extension. TSH, thyroid stimulating hormone; TGAb, thyroglobulin antibody. aTNM staging was determined by 8th American Joint Cancer Committee Tumor-Node-Metastasis stage system.

### Outcomes

The median of follow-up was 63 months (range: 24–122 months). Before initial RAI ablation, 356 patients had no functional or structural evidence of disease and 48 (13.5%) patients had poor prognosis at final follow-up. In addition, 71 patients had highly suspected structural evidence of disease upon ultrasonic examination, and 43 (60.6%) patients had persistent/recurrent disease. Functional evidence of disease was found in 83 patients upon 131I post-therapy WBS, only 16 (19.3%) patients had persistent/recurrent disease, 33 patients had structural and functional evidence of disease, and 22 (66.7%) patients had persistent/recurrent disease.

Further, 442 patients taken one RAI ablation, and 57 (12.9%) patients had poor prognosis. Ninety-two patients taken two RAI ablations, 62 (70.7%) patients had poor prognosis, 9 patients taken three RAI ablations, and 7 (77.8%) patients had poor prognosis.

Generally, long-term remission was obtained in 414 patients (76.2%). In total 129 patients (23.8%) had poor prognosis, 106 patients (19.6%) had a structural incomplete response, and 23 patients (4.2%) had a biochemical incomplete response. Twenty-one patients with positive cytology/histology underwent repeated surgery, 7 patients (33.3%) had a significant effect, 6 patients (28.6%) changed from structural incomplete response to biochemical incomplete response, 7 patients (33.3%) maintained structural incomplete response, and 1 patient (4.8%) had distant metastases. There were no death associated with DTC during study.

### Performance of size of LNM

**Figure 2:**
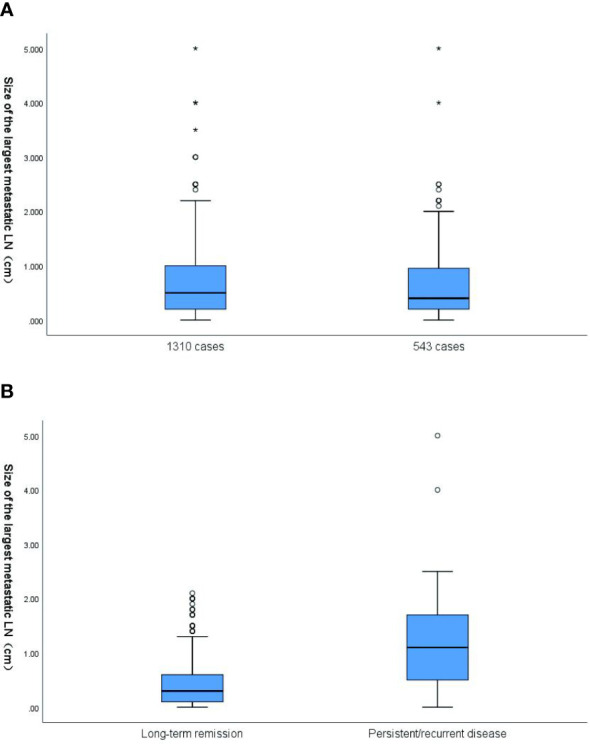
**(A)** Box plot of the distribution of size of the largest metastatic LN in 1310 cases (all patients with size of the largest metastatic LN) and 543 cases (the final cohort consisted of 543 patients). In 1310 cases, the mean value of size of the largest metastatic LN was 0.66cm (range,0-5cm).In 543 cases, the mean value of size of the largest metastatic LN was 0.62cm (range,0-5cm) (p=0.189). **(B)** Box plot of the distribution of size of the largest metastatic LN based on response to the therapy at the end of follow-up. The corresponding median size of the largest metastatic LN was 0.3cm and 1.1cm, respectively, for patients classified as disease free and these with persistent/recurrent disease. The size of the largest metastatic LN results are plotted on a logarithmic scale. LN, lymph node.

The median value of the size of LNM was 0.4 cm (range, 0–5.0 cm), with a median value of 0.3 cm in patients with long-term remission and 1.1 cm in patients with poor prognosis (P < 0.001) (**Figure 2**). The median of time interval among first operation and initial RAI ablation was 2.0 months (range, 0–103 months).

**Figure 3:**
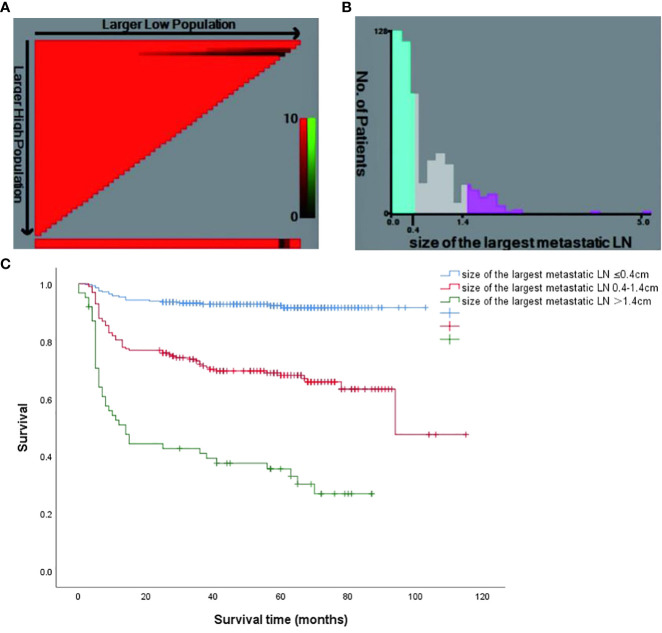
**(A)** The X-tile software of size of the largest metastatic LN for the prediction of persistent/recurrent disease vs long-term remission. **(B)** Patient frequency according to the size of the largest metastatic LN (the optimal cutoffs were 0.4cm and 1.4cm); **(C)** Kaplan Meier survival curves for different risk groups (P < 0.001).

The optimum cut-off value for the size of LNM was decided using the X-tile software (version 3.6.1, Yale University), and the result is shown in **Figure 3A**. All patients were divided into 3 risk stratification for the assessment of long-term remission. There were 282 patients with the size of LNM ≤ 0.4 cm (low-risk group), 199 patients with the size of LNM ranging from 0.4 cm to 1.4 cm (intermediate-risk group), and 62 patients with the size of LNM > 1.4 cm (high-risk group) (**Figure 3B**). Curves of DFS were shown in the Kaplan-Meier method, the low-risk group had 92.2% DFS rates, intermediate-risk group had 67.3% DFS rates, high-risk group had 32.3% DFS rates. The hazard ratio (95% CI) of the high-risk patients and intermediate-risk patients were 13.653 (8.135–22.913) and 4.674 (2.881–7.583), respectively. The low-risk patients was used as the reference (**Figure 3C**).

### Univariate and multivariate analyses of long-term remission

During the univariate analyses, various risk factors were identified to be significantly related to predictors of long-term remission (**Table 2**). Meanwhile, these risk factors were further included in multivariate analysis. Cumulative 131I-administered activity, the size of LNM and ps-Tg were independent factors in predicting clinical outcome (**Table 3**).

**Table 2:** Univariate analysis of predictors of persistent/recurrent disease.

Parameters	Long-term remission	Persistence/recurrence	χ2 or Fisher	P
No. of patients (%	414	129		
Sex Male Female	135 279	56 73	5.033	0.025
Age (y) ≥55 <55	46 368	16 113	0.162	0.687
Extent of lymph node dissection Central lymph node dissection Lateral lymph node dissection Central and lateral lymph node dissection	214 12 188	33 7 89	27.257	<0.001
Number of surgeries 1 2 or moer	384 30	99 30	25.647	<0.001
Histology Classic PTC FV-PTC FTC Aggressive histology	398 8 5 3	121 1 0 7	14.215	0.003
Incomplete tumor resection Yes No	5 409	3 126	0.847	0.357
Gross ETE Yes No	54 360	33 96	11.491	0.001
BMI(kg/m^2^) <18.5 18.5-24 >24	42 214 148	10 73 42	1.321	0.517
Primary tumor size (cm) ≤2 >2 X	304 95 2	86 41 0	4.254	0.119
Bilaterality Yes No	129 285	48 81	1.638	0.201
Multifocality Yes No	178 236	75 54	9.065	0.003
Capsular invasion Yes No	224 190	101 28	23.948	<0.001
Invasion of loco-regional tissues or structures Yes No	83 331	50 79	18.619	<0.001
Vascular invasion Yes No	39 375	25 104	9.383	0.002
Invade nerves Yes No	26 386	15 114	3.966	0.046
T T0T1、T2 T3、T4、Tx	339 62	87 41	16.990	<0.001
N N0 N1a N1b	45 211 158	3 41 85	32.818	<0.001
ATA risk stratification Low Intermediate High	62 309 43	2 96 31	28.478	<0.001
BRAFV600E mutation of primary tumor Yes No	276 68	90 19	0.291	0.589
Number of invaded lymph nodes ≤5 >5	275 139	48 81	34.834	<0.001
Size of the largest metastatic LN(cm) ≤0.4 0.4-1.4 >1.4	260 134 20	22 65 42	114.573	<0.001
Extranodal extension of LN Yes No	123 199	76 34	31.493	<0.001
Cumulative ^131^I-administered activities(GBq) ≤ 7.40 > 7.40	385 29	55 74	162.279	<0.001
ps-Tg (ng/mL) ≤10.1 >10.1	324 90	36 93	111.610	<0.001

ATA, American Thyroid Association; PTC, papillary thyroid cancer; FTC, follicular thyroid cancer; FV-PTC, follicular variant PTC; ps-Tg, preablation stimulated thyroglobulin; LN, lymph node; BMI, Body Mass Index; ETE, extrathyroidal extension.

**Table 3:** Multivariate analysis of predictors of persistent/recurrent disease.

Parameters	OR(95%)	P
Sex Male Female	1 0.993(0.531-1.858)	0.982
Extent of lymph node dissection Central lymph node dissection Lateral lymph node dissection Central and lateral lymph node dissection	1 0.649(0.121-3.482) 0.582(0.175-1.935)	0.614 0.377
Number of surgeries 1 2 or more	1 1.598(0.641-3.983)	0.315
Histology Classic PTC FV-PTC FTC Aggressive histology	1 0.979(0.081-11.846) 0 6.217(0.426-90.734)	0.987 0.999 0.182
Gross ETE Yes No	1 0.950(0.210-4.292)	0.946
Multifocality Yes No	1 1.224(0.649-2.308)	0.533
Capsular invasion Yes No	1 1.165(0.535-2.537)	0.701
Invasion of loco-regional tissues or structures Yes No	1 1.501(0.679-3.319)	0.316
Vascular invasion Yes No	1 1.218(0.485-3.058)	0.674
T T0、T1、T2 T3、T4、Tx	1 0.933(0.339-2.571)	0.894
N N0 N1a N1b	2.432(0.738-8.012)	0.144
ATA risk stratification Low Intermediate High	1 3.278(0.367-29.235) 3.634(0.237-55.784)	0.288 0.354
Number of invaded lymph nodes ≤5 >5	1 0.760(0.363-1.593)	1 0.468
Size of the largest metastatic LN(cm) ≤0.4 0.4-1.4 >1.4	1 2.444(1.100-5.432) 6.614(2.204-19.850)	0.028 0.001
Extranodal extension of LN Yes No	1 1.280(0.660-2.485)	0.465
Cumulative 131I-administered activities(GBq) ≤7,40 >7.40	1 6.679(3.162-14.109)	<0.001
ps-Tg (ng/mL) ≤10.1 >10.1	1 4.646(2.457-8.786)	<0.001

ATA, American Thyroid Association; PTC, papillary thyroid cancer; FTC, follicular thyroid cancer; FV-PTC, follicular variant PTC; ps-Tg, pre-ablation stimulated thyroglobulin; LN, lymph node; BMI, Body Mass Index; ETE, extrathyroidal extension.

### Integrating the size of LNM with ps-Tg

**Figure 4:**
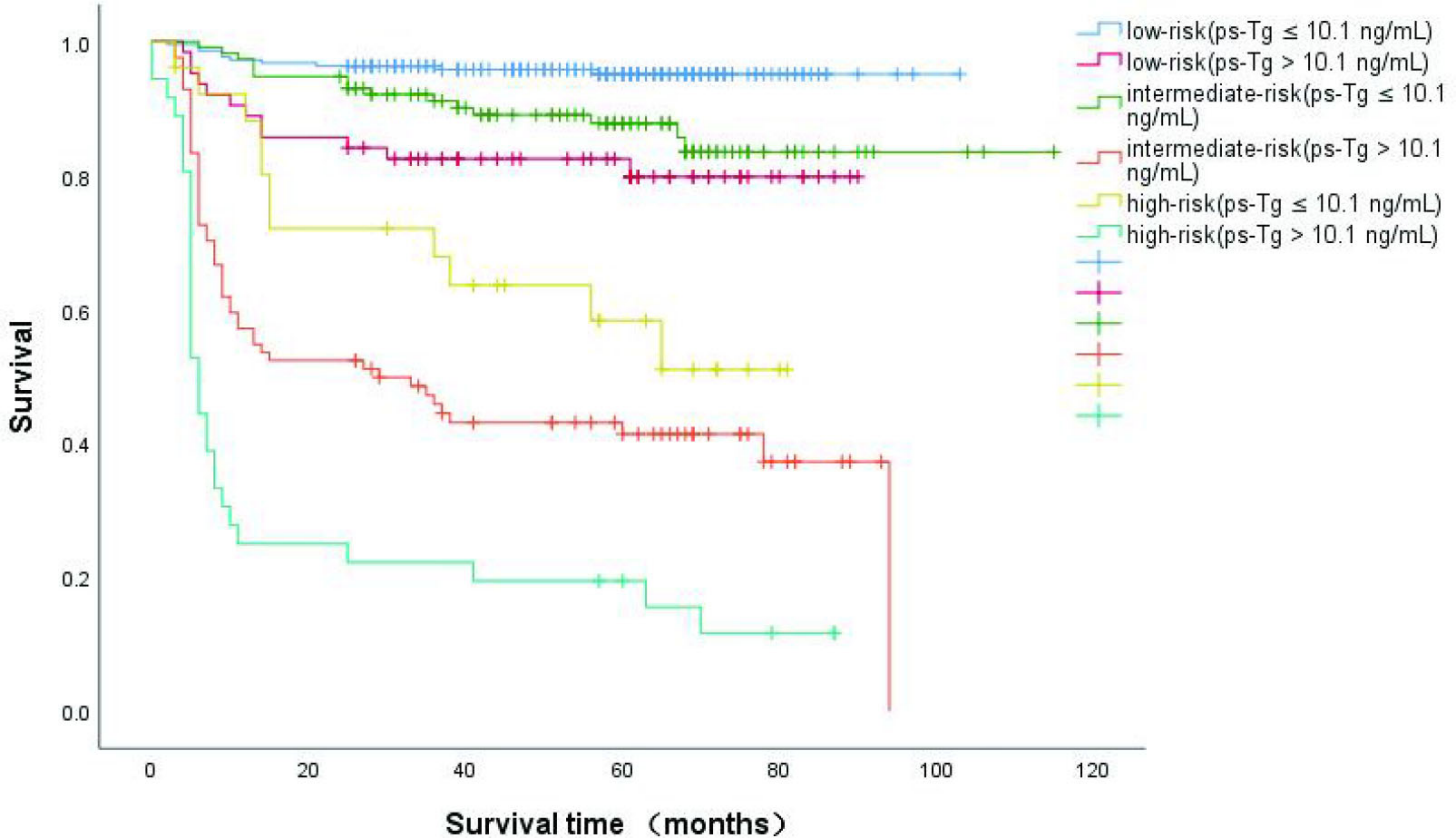
Kaplan-Meier curves for disease-free survival (DFS) in DTC patients according to size of the largest metastatic LN and the integration with ps-Tg,which were measured after thyroid hormone withdrawal.

Recently, some studies report that the ps-Tg cut-off was 10.1 μg/L (13, 16). When we combined the size of LNM with ps-Tg (**Figure 4**), we found that patients with a ps-Tg ≤ 10.1 μg/L had a considerably increased DFS rates (low-risk = 95.4%, intermediate-risk = 87.0%, and high-risk = 57.7%). In contrast, patients with ps-Tg > 10.1 μg/L had a considerably reduced DFS rates in each risk group (92.2–81.0% in low-risk patients, 67.3%–40.5% in intermediate risk patients, and 32.3%–13.9% in high-risk patients) (**Figure 4**).

## Discussion

In our study, we found that patients with highly suspected structural evidence of disease or both highly suspected structural and functional evidence of disease before initial RAI ablation had poorer long-term remission (39.4% and 33.3%, respectively). In addition, patients with NSD or only functional evidence had excellent long-term remission (86.5% and 80.7%, respectively). Patients with two or more RAI ablations had poorer long-term remission (two ablations = 29.3%, three ablations =22.2%). Patients with structural evidence of disease had improved clinical outcomes (61.9%) following repeated surgery.

In summary, our data show the prognostic value of the size of LNM for predicting long-term remission. The cutoff of 0.4 cm and 1.4 cm for a definition of LNM in pN1 can reclassify risk assessment. In our cohort, the low-risk group had 92.2% DFS rates, intermediate-risk group had 67.3% DFS rates, high-risk group had 32.3% DFS rates. This held true for all patients who underwent neck LN dissection.

More significantly, this study demonstrated that integration of the size of LNM with ps-Tg more accurately predicted long-term remission. The ps-Tg ≤ 10.1 μg/L had a greatly increased DFS rates to 95.4%, 87.0%, and 57.7% in the low-, intermediate-, and high-risk patients, respectively. Conversely, the ps-Tg > 10.1 μg/L had a greatly reduced DFS rates for all categories (92.2%–81.0% in low-risk patients, 67.3%–40.5% in intermediate-risk patients, and 32.3%–13.9% in high-risk patients).

Although LNM related to clinical outcomes has been reported previously. For example, in a study including 3,198 TC patients, Zhang et al. (14) found that N stage was associated with poor prognosis. Patients with NX were associated with the worst long-term remission and the highest mortality risk, while patients with pN1 were more possible to occur lung metastasis (48%). In most studies analyzing the size of LNM (cut-off value between 0.2 cm and 3 cm) using the 2015 ATA guidelines, LN micrometastasis (< 0.2 cm) referred to breast cancer and other solid tumors (5, 7, 8, 14, 15, 23). In a study, 16-year follow-up of 233 PTC patients showed that patients with N0 or LN micrometastasis had excellent long-term remission (87.3% and 83.8%, respectively) (10). This is consistent with our findings that patients with N0 and LN micrometastasis had excellent long-term remission (93.9% and 94.9%, respectively), meanwhile, this study found that a size of LNM ≥ 2 mm was significantly associated with persistent/recurrent disease. Differently, Lee et al. (24) found that the newly proposed definition of LN micrometastasis using a cut-off value of 3.5 mm resulted in reclassification of the risk estimates of structural recurrence. And another study has similar report that the size of LNM (cut-off value of 0.536 cm) was significantly associated with a structural incomplete response (7). In addition, several studies have estimated optimal cut-off values (1.0 cm, 1.5 cm, and 3.0 cm, respectively) for the size of LNM to predict clinical outcomes (8, 12). It should be mentioned that the cut-off values (0.4 cm and 1.4 cm) in our study are close to the above results. Finally, in clinical practice, it is very rare to identify bulky LN metastasis (≥3 cm). In a study of 184 patients, Iglesias et al. identified only four cases (2.2%) of huge LN metastasis, which is consistent with the two cases shown in our data (0.4%) (12). Therefore, size of LNM using 2015 ATA guidelines may lead to the inaccurate prediction of clinical outcomes. Furthermore, Barres et al. and Tian et al. (13, 16) demonstrated that ps-Tg (cut-off value of 10.1 ug/L) had significant effects on long-term remission in two large studies involving 1,319 and 2,524 patients. Although the impact of the size of LNM on long-term remission has been reported previously, most studies report only one cut-off value. We divided the patients into 3 risk stratification, and, for the first time, combined the size of LNM with ps-Tg to predict long-term remission.

Some patients did not exhibit LN micrometastasis in the neck preoperative, or were diagnosed as low-risk and did not undergo neck LN dissection (25). This may lead to selection bias, and therefore, such patients were not included in this study (p = 0.13). Although some studies (8, 9) have suggested that having cN0 or LN micrometastasis may not reduce the overall survival rate, Zhao et al. (26) found that prophylactic central neck dissection reduced regional recurrence. Therefore, it has been suggested in recent years that patients with DTC should be offered more aggressive surgery (5, 25, 27).

The overall DFS rate of 76.2% are coincided with the Statistical data found in some recent research (13, 16, 28, 29). However, our research has some shortages. First, different surgical scope, the retrospective design, and the single-center research design were prone to selection biases. Second, the usefulness of the size of LNM and ps-Tg measurements was researched through post analysis. Furthermore, the size of LNM was measured at our hospital before initial RAI ablation in some patients, which may affect the accuracy of measurement. In addition, only the size of LNM and extranodal extension of LN (some data on extranodal extension of LN were missing) were considered. Therefore, we did not analyze each LN and its distribution. Finally, although a median follow-up period of 63 months is an acceptable start for prediction of long-term remission, longer follow-up are required to evaluate the long-term efficacy. However, it is important to note that recurrence disease was far more rare than persistent disease. This may be due to residual lesions (41.5%) caused by surgery (29–31).

## Conclusions

This study suggested the cutoff of 0.4 cm and 1.4 cm for a definition of size of LNM in DTC patients without distant metastases can reclassify risk assessment, and incorporating ps-Tg could more effectively predict clinical outcomes and modify the postoperative management plan.

## Data availability statement

The raw data supporting the conclusions of this article will be made available by the authors, without undue reservation.

## Author contributions

JX and WO contributed to the conception and design of the study. JW, XX, XL and CL organized the database and acquired the data. PC and JQW prepared the figures and tables. JL and LP performed the statistical analysis. JX wrote the first draft of the manuscript. WO and HF critically revised the manuscript and supplemented the data for the final draft of the manuscript. All authors contributed to the article and approved the submitted version.

## References

[1] Lee YC Na SY Park GC Han JH Kim SW Eun YG. Occult lymph node metastasis and risk of regional recurrence in papillary thyroid cancer after bilateral prophylactic central neck dissection: A multi-institutional study. Surgery (2017) 161(2):465–71. doi: 10.1016/j.surg.2016.07.031 27574773

[2] Feng JW Yang XH Wu BQ Sun DL Jiang Y Qu Z. Predictive factors for central lymph node and lateral cervical lymph node metastases in papillary thyroid carcinoma. Clin Transl Oncol (2019) 21(11):1482–91. doi: 10.1007/s12094-019-02076-0 30879178

[3] Liu C Xiao C Chen J Li X Feng Z Gao Q. Risk factor analysis for predicting cervical lymph node metastasis in papillary thyroid carcinoma: A study of 966 patients. BMC Canc. (2019) 19(1):622. doi: 10.1186/s12885-019-5835-6 PMC6593593 31238891

[4] Xu SY Yao JJ Zhou W Chen L Zhan WW. Clinical characteristics and ultrasonographic features for predicting central lymph node metastasis in clinically node-negative papillary thyroid carcinoma without capsule invasion. Head Neck. (2019) 41(11):3984–91. doi: 10.1002/hed.25941 31463972

[5] Haugen BR Alexander EK Bible KC Doherty GM Mandel SJ Nikiforov YE. 2015 American Thyroid association management guidelines for adult patients with thyroid nodules and differentiated thyroid cancer: The American thyroid association guidelines task force on thyroid nodules and differentiated thyroid cancer. Thyroid (2016) 26(1):1–133. doi: 10.1089/thy.2015.0020 26462967 PMC4739132

[6] Grani G Zatelli MC Alfò M Montesano T Torlontano M Morelli S. Real-world performance of the American thyroid association risk estimates in predicting 1-year differentiated thyroid cancer outcomes: A prospective multicenter study of 2000 patients. Thyroid (2021) 31(2):264–71. doi: 10.1089/thy.2020.0272 32475305

[7] Deng Y Zhu G Ouyang W Pan L Feng H Wu J. Size of the largest metastatic focus to the lymph node is associated with incomplete response of pn1 papillary thyroid carcinoma. Endocr Pract (2019) 25(9):887–98. doi: 10.4158/EP-2018-0583 31170371

[8] Randolph GW Duh QY Heller KS LiVolsi VA Mandel SJ Steward DL. The prognostic significance of nodal metastases from papillary thyroid carcinoma can be stratified based on the size and number of metastatic lymph nodes, as well as the presence of extranodal extension. Thyroid (2012) 22(11):1144–52. doi: 10.1089/thy.2012.0043 23083442

[9] Liu W Yan X Dong Z Su Y Ma Y Zhang J. A mathematical model to assess the effect of residual positive lymph nodes on the survival of patients with papillary thyroid microcarcinoma. Front Oncol (2022) 12:855830. doi: 10.3389/fonc.2022.855830 35847961 PMC9279734

[10] Kim SY Kim BW Pyo JY Hong SW Chang HS Park CS. Macrometastasis in papillary thyroid cancer patients is associated with higher recurrence in lateral neck nodes. World J Surg (2018) 42(1):123–9. doi: 10.1007/s00268-017-4158-5 28779384

[11] Leboulleux S Girard E Rose M Travagli JP Sabbah N Caillou B. Ultrasound criteria of malignancy for cervical lymph nodes in patients followed up for differentiated thyroid cancer. J Clin Endocrinol Metab (2007) 92(9):3590–4. doi: 10.1210/jc.2007-0444 17609301

[12] Iglesias C González O Temprana-Salvador J García-Burillo A Caubet E Ramón YCS. Nodal metastatic load in papillary thyroid carcinoma. morphological and molecular analysis with one-step nucleic acid amplification on more than 550 lymph nodes. Endocrinol Diabetes Nutr (Engl Ed). (2021) 68(5):346–53. doi: 10.1016/j.endinu.2020.04.004 32800751

[13] Barres B Kelly A Kwiatkowski F Batisse-Lignier M Fouilhoux G Aubert B. Stimulated thyroglobulin and thyroglobulin reduction index predict excellent response in differentiated thyroid cancers. J Clin Endocrinol Metab (2019) 104(8):3462–72. doi: 10.1210/jc.2018-02680 30785995

[14] Zhang J Cheng X Shen L Wang X Wang L Sun X. The association between lymph node stage and clinical prognosis in thyroid cancer. Front Endocrinol (Lausanne). (2020) 11:90. doi: 10.3389/fendo.2020.00090 32174889 PMC7056822

[15] Jeon MJ Kim M Park S Oh HS Kim TY Kim WB. A follow-up strategy for patients with an excellent response to initial therapy for differentiated thyroid carcinoma: Less is better. Thyroid (2018) 28(2):187–92. doi: 10.1089/thy.2017.0130 29179642

[16] Tian T Xu Y Zhang X Liu B. Prognostic implications of preablation stimulated tg: A retrospective analysis of 2500 thyroid cancer patients. J Clin Endocrinol Metab (2021) 106(11):e4688–e97. doi: 10.1210/clinem/dgab445 34143886

[17] Pan L Chen Y Li S Ouyang W Feng H Wu J. Postoperative thyroid remnants for differentiated thyroid cancer may not affect the outcome of high-dose radioiodine therapy. Oral Oncol (2020) 104:104610. doi: 10.1016/j.oraloncology.2020.104610 32143113

[18] Cooper DS Doherty GM Haugen BR Kloos RT Lee SL Mandel SJ. Revised American thyroid association management guidelines for patients with thyroid nodules and differentiated thyroid cancer. Thyroid (2009) 19(11):1167–214. doi: 10.1089/thy.2009.0110 19860577

[19] Aydin C Dellal FD Tam AA Ogmen B Kilicarslan A Topaloglu O. Comparative analysis of diagnostic adequacy rate between aspiration and nonaspiration techniques of fine-needle cytology in patients with thyroid cancer and ultrasonographically suspicious cervical lymph nodes. Diagn Cytopathol. (2017) 45(10):889–94. doi: 10.1002/dc.23793 28834301

[20] Mitchell AL Gandhi A Scott-Coombes D Perros P. Management of thyroid cancer: United kingdom national multidisciplinary guidelines. J Laryngol Otol (2016) 130(S2):S150–s60. doi: 10.1017/S0022215116000578 PMC4873931 27841128

[21] Machado MR Tavares MR Buchpiguel CA Chammas MC. Ultrasonographic evaluation of cervical lymph nodes in thyroid cancer. Otolaryngol Head Neck Surg (2017) 156(2):263–71. doi: 10.1177/0194599816676472 28145839

[22] Wang Z Tang C Wang Y Yin Z Rixiati Y. Inclusion of the number of metastatic lymph nodes in the staging system for medullary thyroid cancer: Validating a modified American joint committee on cancer tumor-Node-Metastasis staging system. Thyroid (2022) 32(5):536–43. doi: 10.1089/thy.2021.0571 35350868

[23] Cranshaw IM Carnaille B. Micrometastases in thyroid cancer. import. finding? Surg Oncol (2008) 17(3):253–8. doi: 10.1016/j.suronc.2008.04.005 18504121

[24] Lee YM Park JH Cho JW Hong SJ Yoon JH. The definition of lymph node micrometastases in pathologic N1a papillary thyroid carcinoma should be revised. Surgery (2019) 165(3):652–6. doi: 10.1016/j.surg.2018.09.015 30385127

[25] Schlumberger M Leboulleux S. Current practice in patients with differentiated thyroid cancer. Nat Rev Endocrinol (2021) 17(3):176–88. doi: 10.1038/s41574-020-00448-z 33339988

[26] Zhao W You L Hou X Chen S Ren X Chen G. The effect of prophylactic central neck dissection on locoregional recurrence in papillary thyroid cancer after total thyroidectomy: A systematic review and meta-analysis : pCND for the locoregional recurrence of papillary thyroid cancer. Ann Surg Oncol (2017) 24(8):2189–98. doi: 10.1245/s10434-016-5691-4 27913945

[27] Eltelety AM Terris DJ. Neck dissection in the surgical treatment of thyroid cancer. Endocrinol Metab Clin North Am (2019) 48(1):143–51. doi: 10.1016/j.ecl.2018.11.004 30717898

[28] Jayasekara J Jonker P Lin JF Engelsman AF Wong MS Kruijff S. Early postoperative stimulated serum thyroglobulin quantifies risk of recurrence in papillary thyroid cancer. Surgery (2020) 167(1):40–5. doi: 10.1016/j.surg.2019.06.048 31515121

[29] Sapuppo G Tavarelli M Belfiore A Vigneri R Pellegriti G. Time to separate persistent from recurrent differentiated thyroid cancer: Different conditions with different outcomes. J Clin Endocrinol Metab (2019) 104(2):258–65. doi: 10.1210/jc.2018-01383 30165559

[30] Miller JE Al-Attar NC Brown OH Shaughness GG Rosculet NP Avram AM. Location and causation of residual lymph node metastasis after surgical treatment of regionally advanced differentiated thyroid cancer. Thyroid (2018) 28(5):593–600. doi: 10.1089/thy.2017.0434 29562827

[31] Bates MF Lamas MR Randle RW Long KL Pitt SC Schneider DF. Back so soon? is early recurrence of papillary thyroid cancer really just persistent disease? Surgery (2018) 163(1):118–23. doi: 10.1016/j.surg.2017.05.028 PMC5736421 29128176

