# Corrigendum: Incorporation size of lymph node metastasis focus and pre-ablation stimulated Tg could more effectively predict clinical outcomes in differentiated thyroid cancer patients without distant metastases

**DOI:** 10.3389/fendo.2023.1221004

**Published:** 2023-07-14

**Authors:** Jiahao Xie, Pan Chen, Jing Wang, Xiaoqin Luo, Jiaxin Luo, Xiaoli Xiong, Chunyan Li, Liqin Pan, Juqing Wu, Huijuan Feng, Wei Ouyang

**Affiliations:** Department of Nuclear Medicine, The Zhujiang Hospital, Southern Medical University, Guangzhou, Guangdong, China

**Keywords:** differentiated thyroid cancer, lymph node metastasis, pre-ablation stimulated Tg, 131I therapy, prognosis

In the published article, there was an error in **Figure 3** as published. The third legend in **Figure 3C** incorrectly stated “size of the largest metastatic LN>0.4cm”. The corrected **Figure 3** and its caption appear below.

**Figure 3:**
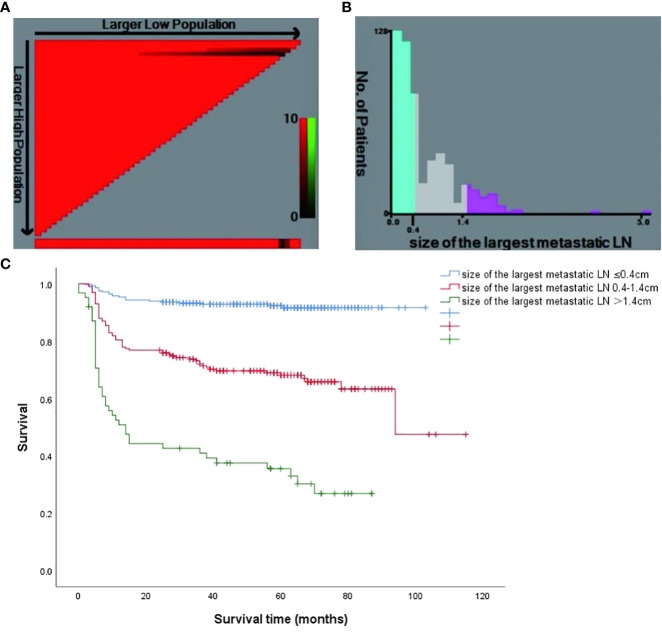
**(A)** The X-tile software of size of the largest metastatic LN for the prediction of persistent/recurrent disease vs long-term remission. **(B)** Patient frequency according to the size of the largest metastatic LN (the optimal cutoffs were 0.4cm and 1.4cm); **(C)** Kaplan Meier survival curves for different risk groups (P < 0.001).

In the published article, there was an error. The decimals in Tg cutoff values were incorrect throughout the text. Instead of “10.0”, it should be “10.1”.

The authors apologize for these errors and state that these do not change the scientific conclusions of the article in any way. The original article has been updated.

